# Reduction-responsive immobilised and protected enzymes†

**DOI:** 10.1039/d4na00580e

**Published:** 2024-11-28

**Authors:** Congyu Wu, Seyed Amirabbas Nazemi, Natascha Santacroce, Jenny A. Sahlin, Laura Suter-Dick, Patrick Shahgaldian

**Affiliations:** a School of Life Science, University of Applied Sciences and Arts Northwestern Switzerland Hofackerstrasse 30, Muttenz CH-4132 Switzerland patrick.shahgaldian@fhnw.ch laura.suterdick@fhnw.ch; b Swiss Center for Applied Human Toxicology (SCAHT) Missionsstrasse 64 Basel CH-4055 Switzerland; c Swiss Nanoscience Institute Klingelbergstrasse 82 Basel CH-4056 Switzerland

## Abstract

We report a synthetic strategy to produce nano-immobilised and organosilica-shielded enzymes of which the biocatalytic activity is, by design, chemically enhanced under reductive conditions. The enzymes were immobilised onto silica nanoparticles through a reduction-responsive crosslinker and further shielded in an organosilica layer of controlled thickness. Under reducing conditions, disulphide bonds linking the protein to the carrier material were reduced, triggering enzyme activation. The organosilica shield prevents the enzymes from leaching from the nanobiocatalysts and preserves their integrity.

## Abstract 2

Enzymes unchained – enzymes immobilised on nanoparticles with a cleavable linker and shielded in an organosilica layer are activated upon reductive breakage of the linker. The organosilica layer prevents enzyme leaching.

## Graphical abstract

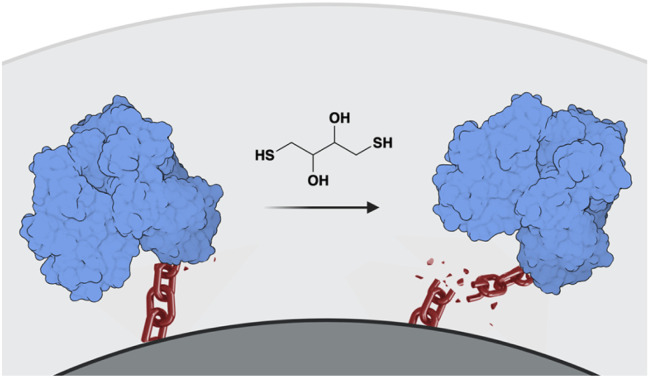

The development of stimuli-responsive materials is an active field of research as it opens great perspectives in a broad range of applications including electronics, soft robotics, (bio)sensing, diagnostics and biomedicine.^1–7^ Such materials are designed to change their chemical or physical properties upon an external trigger. For biomedical applications, a vast range of drug delivery vehicles have been designed to respond to a variety of physiologically relevant endogenous stimuli such as specific enzymes (*e.g.*, matrix metalloproteinases, serum proteases, cathepsin), pH, glucose, hypoxia, ATP and redox signals.^1,5,6^ Regarding the latter, it is established that cancer tissue microenvironments display concentrations of glutathione (GSH), a tripeptide involved in maintaining cellular homeostasis and redox balance, higher than their healthy counterpart.^8^ Scientists have exploited this phenomenon to produce a variety of drug transport systems sensitive to the disulphide-reducing properties of GSH. They include mesoporous silica,^9–11^ hydrogels,^12,13^ polymers,^14–16^ gold nanoparticles^17^ and nanorods.^18^ While the development of delivery systems for stimuli-responsive chemotherapeutics have mainly focused on small organic drugs, the delivery of biologics has also been studied. For example, Katz *et al.* have recently reported on the delivery of monoclonal antibodies using magnetic responsive microgel nanocomposite.^19^ While a variety of pathologies can be treated with enzymes, including metabolic deficiencies, ocular diseases, joint pathologies, and cancer, their stimuli-responsive activation has been mainly studied in the context of biocatalytic production.^20^ For example, enzymes immobilised on a variety of surfaces (*e.g.*, hydrogels, magnetic^21^ or plasmonic^22^ nanoparticles, carbon nanotubes^23^) can be activated using stimuli such as light^22,23^ or a magnetic field.^21^ We previously reported a method of enzyme immobilisation and protection using silica nanoparticles as carriers, and ultrathin organosilica layers of controlled composition and thickness as enzyme shields.^24–29^ This strategy allowed the development of plasmonic photothermally activable nanobiocatalysts.^24^

Herein, we report a method of redox-responsive enzyme activation. It exploits the loss of activity experienced by enzymes when immobilised on solid supports, due to loss of conformational mobility. This method is based on the immobilisation of an enzyme, at the surface of silica particles (SNPs), using a disulphide-containing bifunctional crosslinker followed by the growth of an organosilica layer at the surface of the SNPs. The chemical reduction of disulphide bonds is expected to increase the enzyme's conformational mobility, resulting in its activation while the shield prevents its release; Fig. 1.

**Fig. 1:**
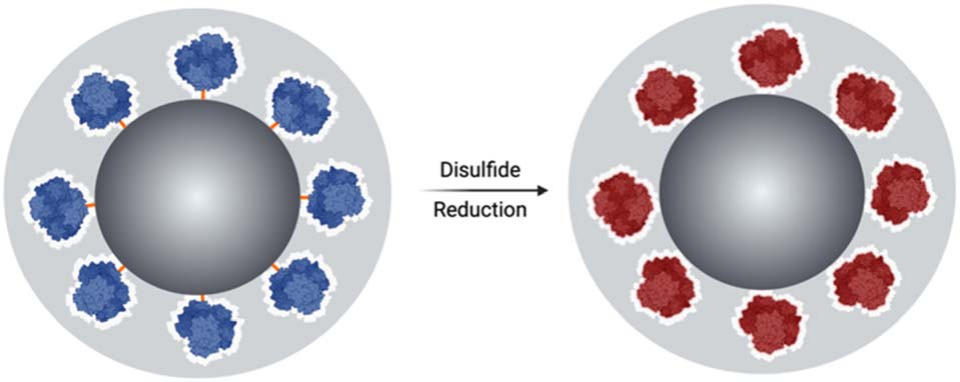
The schematic figure illustrates the breaking of the crosslinker DSP and the subsequent release of the enzyme, which is shielded within the organosilica layer and activated upon redox stimuli triggering.

As carrier material, we used amine-bearing silica particles (SNP–NH_2_) produced using a modified Stöber method^30^ yielding non-porous SNPs.^31^ This was followed by amino-modification using (3-aminopropyl)triethoxysilane (Fig. 2).^28^ The particles produced were characterised by means of scanning electron microscopy (SEM, and statistical image analysis) that revealed smooth and spherical particles with an average diameter of 226 ± 8 nm and a polydispersity index of 0.03 (Fig. 3). As a model enzyme, we selected a widely studied hydrolase enzyme, namely β-galactosidase (βgal) from *K. lactis*. It is a globular protein that can be approximated as triaxial ellipsoid with dimensions 15.9 × 9.3 × 5.3 nm (3OB8). SNP–NH_2_ were further reacted with a cleavable bifunctional crosslinker, namely 3,3′-dithiodipropionic acid di(*N*-hydroxysuccinimide ester) (DSP) and subsequently with βgal (180 μg mL^−1^ in 10 mM MES buffer, 5 mM MgCl_2_, pH 6.2) at 20 °C for 30 min, yielding SNP–DSP–βgal. To determine the enzyme immobilisation yield, a protein quantification assay was performed on the liquid fraction of the reaction mixture, collected by centrifugation after the bioconjugation reaction. The immobilisation amount reached 29.8 μg of βgal per mg of SNP–NH_2_, corresponding to an immobilisation yield of 52%. We also produced a control system (SNP–Glu–βgal) produced with a non-cleavable crosslinker, namely glutaraldehyde, following a method previously reported.^22^ In order to verify the possibility to reductively cleave the crosslinker and to release the immobilised enzyme, SNP–DSP–βgal were reacted with a reducing agent, namely dithiothreitol (DTT, 50 mM), at 20 °C; the soluble fraction of the reaction mixture was analysed by means of sodium-dodecyl-sulphate polyacrylamide gel electrophoresis (SDS-PAGE) (Fig. S2†). The results showed that 91% of the enzyme was released after this treatment. In contrast, no enzyme release was observed in the groups of SNP–Glu–βgal. These results confirmed the possibility to release the enzyme upon reduction of the disulphide crosslinker. Next, immobilised βgal systems using either DSP or glutaraldehyde, hereafter referred to as SNP–DSP–βgal and SNP–Glu–βgal, respectively, were shielded within an organosilica (OS) layer. This is expected to provide protection to the immobilised enzyme and prevents its release upon cleavage of the DSP crosslinker. The size of βgal, a large tetrameric protein, required a layer of at least 16 nm in thickness to fully cover the enzyme.^28^ This was achieved by reacting the immobilised enzymes with tetraethylorthosilicate (TEOS) and APTES in ammonium bicarbonate buffer (10 mM, pH 7.8) at 10 °C, which yielded SNP–DSP–βgal–OS and SNP–Glu–βgal–OS, respectively. During the layer growth reaction, sample aliquots were collected at increasing reaction durations; the particles produced were characterised by SEM; Fig. 3 and S1.† SEM characterisation confirmed the successful layer growth reaction on SNP–DSP–βgal–AT, with average layer thickness values of 4, 11, 14 and 17 nm for reaction durations of 20, 40, 60 and 80 min, respectively. The presence of the protective layer is observed on all particles; this confirmed the lack of SNPs aggregation during the layer growth reaction. While the surface of the particles appeared to be slightly rougher, the dispersity remained mainly unchanged with a polydispersity index of 0.04 after 80 min reaction. The morphology, size distribution, and layer growth kinetics of SNP–Glu–βgal–OS did not show relevant differences from those of SNP–DSP–βgal–OS, indicating that the layer growth process was not affected by the chemical nature of the linker (Fig. S1†). SNP–DSP–βgal–OS were submitted to DTT treatment; analysis of the liquid phase by SDS-PAGE did not show relevant bands corresponding to the released enzyme. This confirmed that the OS shield prevented the enzyme's release (Fig. S2†).

**Fig. 2:**
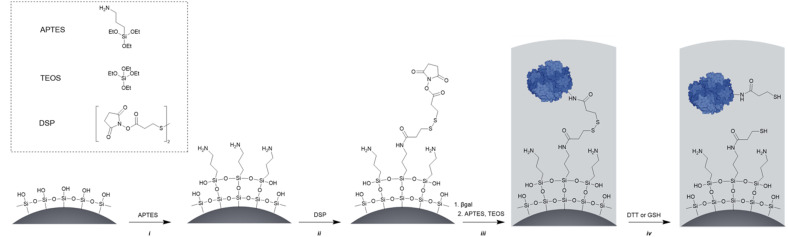
Synthetic route to reduction-responsive immobilised and shielded enzymes. The main synthetic steps are as follows: (i) amino-modification using APTES, (ii) cross linker [dithiobis(succinimidyl propionate), DSP] reaction with amino functions introduced at the surface of the SNPs. The low concentration of SNPs and electrostatic repulsion of SNPs prevent particle cross-linking; (iii) protein surface anchoring *via* the reaction with *N*-hydroxysuccinimide-activated surface and organosilica shield production, and (iv) disulphide bond reduction (using DTT or glutathione) releasing the enzyme within the organosilica shield.

**Fig. 3:**
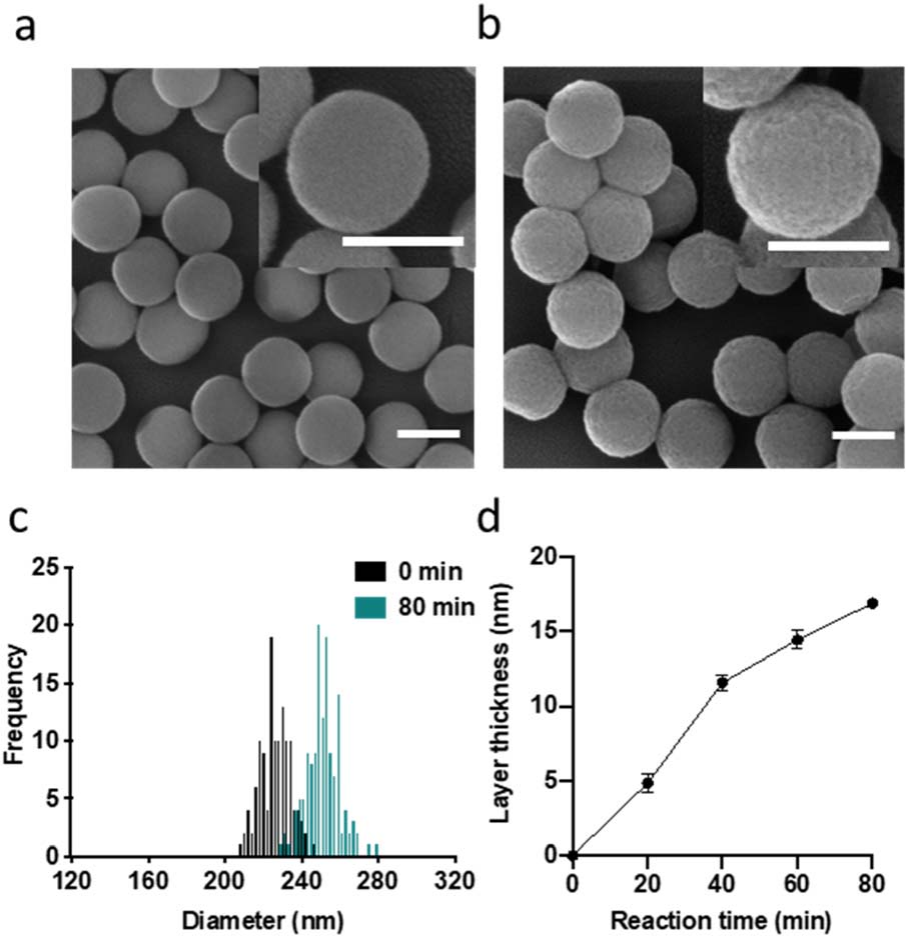
SEM micrographs of SNPs (a) and SNP–DSP–βgal–OS after 80 min of OS layer growth reaction (b). Size distribution (c) and layer growth kinetics (d) measured on SEM micrographs. Every point is the average of at least 100 particles. All scale bars represent 200 nm. Standard error is calculated as 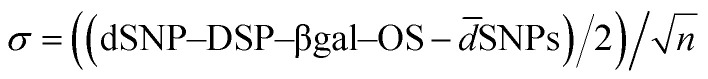 where *n* is the number of measured particles.

The enzymatic activity of the particles produced was evaluated by an established spectrophotometric (*o*-nitrophenyl-β-galactoside, ONPG) assay. The results showed that the activities of immobilised enzyme (SNP–DSP–βgal and SNP–Glu–βgal) were 74 and 72 mU mg^−1^, respectively (Fig. S3†); this is in agreement with previously published results.^28^ They retained 50% and 48% of the activity of the soluble enzyme, respectively. The decrease in activity is probably caused by unfavourable orientations or partial denaturation of the immobilised enzyme. To assess the redox-triggered activation of the enzymatic activity, SNP–DSP–βgal–OS and SNP–Glu–βgal–OS were tested after reaction with DTT (50 mM, 30 min) and thorough washing; Fig. 4. In the control groups without DTT treatment, the activities of SNP–Glu–βgal–AT were 10, 28, 25, and 22 mU mg^−1^ after 20, 40, 60 and 80 min of OS layer growth reaction durations. Similarly, the activities of SNP–DSP–βgal–OS were 11, 17, 15 and 13 mU mg^−1^, respectively. The activities of SNP–Glu–βgal–OS slightly increased by 15%, 18%, 20% and 6% after DTT treatment, compared to the groups of SNP–Glu–βgal–AT with the same layer growth duration. The minor increase in activity in SNP–Glu–βgal–AT is attributed to the modification of cysteine residues in βgal by DTT,^26^ as βgal was immobilised *via* a nonreduction-responsive crosslinker (glutaraldehyde). Interestingly, the biocatalytic activity of SNP–DSP–βgal–AT considerably increased after DTT treatment by 78%, 86%, 58% and 53% for layer thickness values of 4, 11, 14, and 17 nm, respectively. This set of measurements confirmed the reduction-activation of the enzyme and suggested that the enhanced activity of SNP–DSP–βgal–AT was mainly due to increased conformational freedom resulting from the breakage of crosslinker triggered by the reducing agent.

**Fig. 4:**
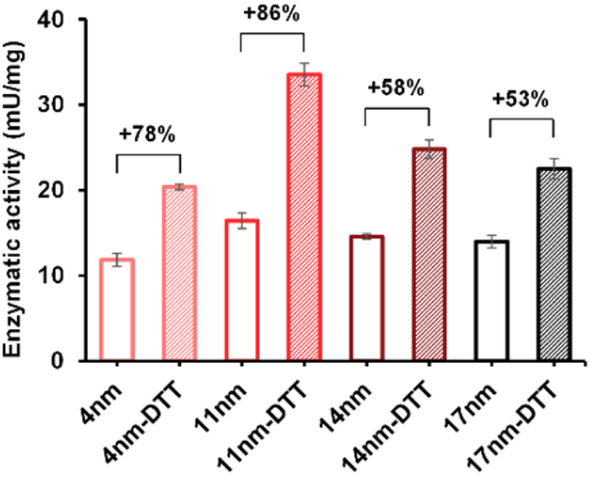
Enzymatic activity of SNP–DSP–βgal–OS with and without DTT treatment. Error bars represent standard deviation measured on triplicates.

To verify the versatility of the strategy developed, we investigated the immobilisation of another relevant enzyme, l-asparaginase (ASNase). It is a bacterial amidase that catalyses the hydrolysis of asparagine into aspartic acid. It is used in oncology for the depletion of plasma l-asparagine to inhibit cancer cells' growth, which have lost the ability to effectively synthesise asparagine. While ASNase is established in paediatric oncology for the treatment of *e.g.* acute lymphoblastic leukaemia, side effects, owing to the immunogenicity of this bacterial enzyme, remain a major limitation of this therapeutic approach. OS shielding can serve as a valuable alternative to overcome ASNase immunogenicity, hiding the enzyme from the immune system. The additional feature of reduction-triggered activation may enhance enzyme cytotoxicity when exposed to the reducing environment of cancer tissues. From a molecular viewpoint, ASNase is a globular protein with an average diameter of 7 nm as measured from the crystal structure of the protein (PDB code: 6V5F).^32^ We immobilised ASNase with DSP on SNP–NH_2_, with an immobilisation of 3.2 μg ASNase per mg of particles, corresponding to an immobilization yield of 96%. Layer growth was performed using the procedure applied with βgal with DSP to yield SNP–DSP–ASNase–OS. Reference particles, SNP–DSP–ASNase–OS, were prepared similarly using glutaraldehyde. The layer growth kinetics were monitored and demonstrated to be comparable to that measured with βgal (Fig. 5a). The activity of the shielded enzyme was monitored using the native substrate, l-Asp, and measuring ammonia formation using the Nessler reagent.^33^ SNP–DSP–ASNase–OS activity measurement showed values of 17, 69, and 78 mU mg^−1^ after 30, 45, and 60 minutes of layer growth reactions. This corresponds to activity retentions of 12, 50, and 57%, respectively. DTT treatment resulted in an increase in activity of 51%, 27%, and 37%, respectively; see Fig. 5b. This confirmed the activation of the enzyme when submitted to reducing conditions. Cancer cells are typically considered to exist in reducing environments, primarily owing to their altered metabolic processes and increased GSH content.^34^ This prompted us to study the GSH activating effect on SNP–DSP–ASNase–OS; Fig. 5. After treating these particles with GSH for 30 minutes, we observed a 16% increase in activity compared to the untreated group, while SNP–Glu–ASNase–AT showed a decrease in activity of 18.5%. Hence, we could demonstrate enzyme activation in the presence of GSH. The moderate increase in activity may be attributed to a concomitant deleterious effect of GSH on the enzyme, as suggested by the decrease in activity of SNP–Glu–ASNase–AT. This was supported by the loss in activity of the soluble enzyme when treated with GSH; Fig. S4.† Next, we examined the cytotoxicity of SNP–DSP–ASNase–AT on cancer cells for which the sensitivity to ASNase activity is established, namely hepatocellular carcinoma HepG2 cells.^35,36^ The cytotoxic effect of SNP–DSP–ASNase–AT was compared to the effect of SNPs and SNP–Glu–ASNase–OS. First, our results showed that SNPs did not significantly affect cell viability up to a concentration as high as 32 mg mL^−1^ (Fig. S5†). Both SNP–DSP–ASNase–AT and SNP–Glu–ASNase–OS caused dose-dependent cell toxicity, which was higher for the reduction-sensitive systems. At an ASNase concentration of 2.1 U mL^−1^, cell viability in the SNP–DSP–ASNase–OS group was only 55%, whereas it was 78.6% in the SNP–Glu–ASNase–OS group.

**Fig. 5:**
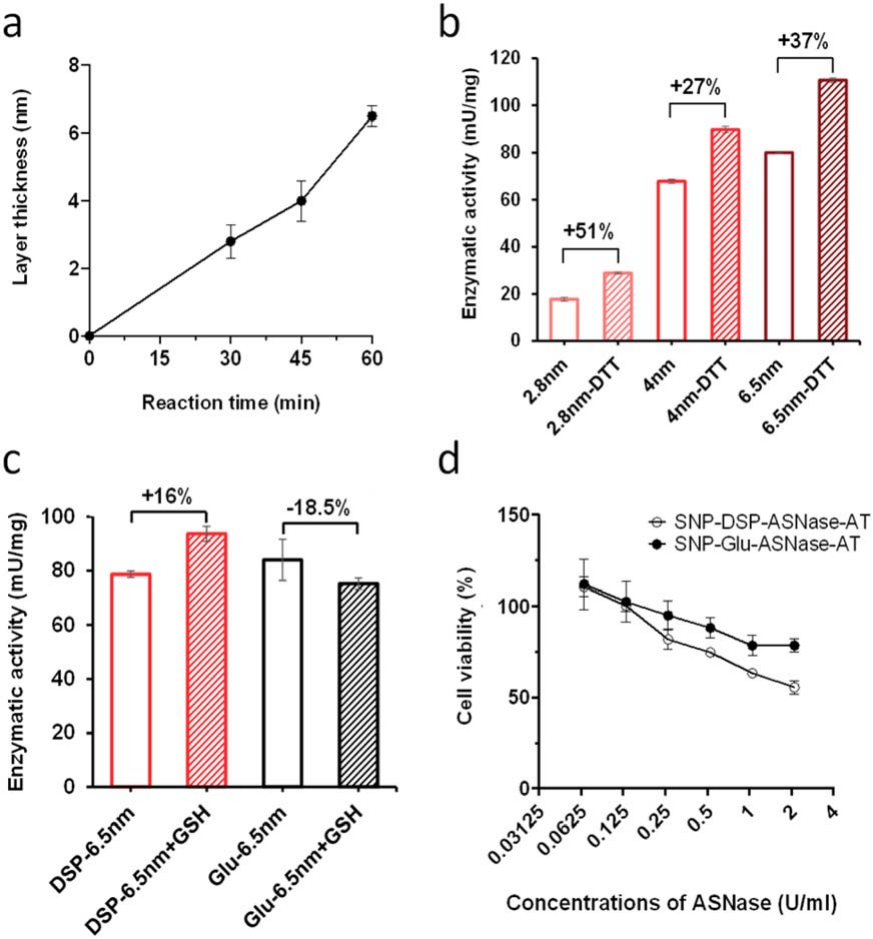
Kinetics of layer growth (a) and enzymatic activity of SNP–DSP–ASNase–AT with and without DTT treatment corresponding to increasing layer thickness (2.8, 4, and 6.5 nm) after 30, 45, and 60 min of reaction, respectively (b). Enzymatic activity of SNPs–DSP–ASNase–AT and SNPs–Glu–ASNase–AT with and without glutathione (GSH) treatment (c). Cytotoxicity of SNPs–DSP–ASNase–AT and SNPs–Glu–ASNase–AT on HepG2 cells (d). Error bars represent standard deviation measured on triplicates.

In summary, our study presents a novel approach to immobilise enzymes through a reduction-responsive crosslinker, followed by shielding in an organosilica layer. Our results suggest that the enzyme conformational mobility is increased by reducing conditions, causing enzymatic activation. The preliminary investigations carried out on cancer cells highlight the potential of this approach for therapeutic purposes.

## Data availability

Data for this article, including [unprocessed SEM micrographs, nanoparticles size distribution tables, enzymatic activity values, cell viability results] are available at Zenodo at https://doi.org/10.5281/zenodo.13319739.

## Conflicts of interest

There are no conflicts to declare.

## Supplementary Material

NA-007-D4NA00580E-s001
