# Supplementary material for: Reduction-responsive immobilised and protected enzymes
Source: Nanoscale Adv. 2024 Nov 28;7(1):89–93. doi: 10.1039/d4na00580e (PMC11603382; doi:10.1039/d4na00580e)
Supplement: NA-007-D4NA00580E-s001 [file NA-007-D4NA00580E-s001.pdf]

Supplementary Information (SI) for Nanoscale Advances.
This journal is © The Royal Society of Chemistry 2024

Electronic Supplementary Information

Reduction-responsive organosilica shielded enzymes

Congyu Wu,[a] Seyed Amirabbas Nazemi,[a] Natascha Santacroce,[a] Jenny A. Sahlin,[a] Laura Suter-Dick*[a,b] and Patrick Shahgaldian*[a,c]

[a] School of Life Science, University of Applied Sciences and Arts Northwestern Switzerland, Hofackerstrasee 30, Muttenz CH-4132, Switzerland.

[b] Swiss Center for Applied Human Toxicology (SCAHT), Basel, Switzerland

[c] Swiss Nanoscience Institute, Klingelbergstrasse 82, Basel CH-4056, Switzerland.

E-mail: patrick.shahgaldian@fhnw.ch, laura.suterdick@fhnw.ch

Materials and methods

Chemicals

Tetraethyl orthosilicate (TEOS, ≥ 99%), (3-aminopropyl)triethoxysilane (APTES, ≥ 98%), ammonium hydroxide (ACS grade, 28–30%), ethanol (ACS grade, anhydrous), dimethyl sulfoxide (anhydrous, ≥ 99.9%), ammonium bicarbonate (≥ 98%), sodium phosphate dibasic (ACS grade, anhydrous, ≥ 99%), trichloroacetic acid solution (TCA, 6.1 N), sodium carbonate (Na_2CO_3, ≥ 99.5%), potassium phosphate monobasic (ACS grade, ≥ 99%), β-galactosidase from kluyveromyces lactis (βgal), asparaginase (ASNase, from *Escherichia coli*), esterase from horse liver (lyophilized), 2-nitrophenol (ONP, 98%), 4-nitrophenol (PNP, 99.9%), 4-nitrophenyl butyrate (98%), glutaraldehyde (Glu, Grade I, 25% in H_2O), and 1,4-dithiothreitol (DTT) were purchased from Sigma-Aldrich. Dithiobis (succinimidyl propionate) (DSP) and potassium phosphate dibasic, potassium phosphate monobasic, Pierce™ 660 nm protein assay kit, and BCA protein quantification kit were purchased from Fisher Scientific. 2-Nitrophenyl-beta-D-galactopyranoside (ONPG, 99%) was purchased from Acros Organics. Bovine serum albumin (BSA, 98%) was purchased from Carl Roth. Spectrum Spectra/Por® 7 membrane tubing (50kDa MWCO) was purchased from Thermo Fisher Scientific. SDS-PAGE gel and Bio-Safe™ Coomassie G-250 Stain were purchased from Bio-Rad.

Buffer solutions

ABC buffer: 10 mM ammonium bicarbonate, pH 7.8. NaPB: 29 mM sodium phosphate dibasic, 21 mM sodium phosphate monobasic, pH 7.0. KPB: 100 mM potassium phosphate monobasic, pH 5.0. 10 mM MES buffer (5 mM $MgCl_2$, pH 6.2).

Equipment

Enzyme activity assays were performed in a Thermomixer (Eppendorf) and monitored using a Synergy H1 spectrometer (BioTek) into a 96-well plate. Nanopure water (resistivity ≥ 18 MΩ-cm) was produced with a Millipore ®Synergy purification system (Merck). Particles were imaged using a Zeiss SUPRA® 40VP scanning electron microscope. Particle size was measured using the Olympus Analysis software package. SDS-PAGE was carried out using a PowerPac™ basic power supply (BioRad). SDS-PAGE gels were stained using a staining solution (Bio-Safe™ Coomassie G-250 Stain) from Bio-Rad.

Experimental procedure

Amino modification of SNPs

Silica nanoparticles (SNPs) were synthesized according to an established procedure described by Cumbo *et al*.[1] In a round-bottom flask, 130 µL of APTES (0.56 mmol) was added to 200 mL of 3.2 mg/mL of SNPs in H_2O, and the mixture was stirred under 400 rpm in a water batch at 20 °C for 1.5 h. Subsequently, the particles were washed with nanopure H_2O, centrifuged at 4000 rpm for 15 min for two cycles. Then the particles produced, SNP-NH_2 were resuspended in water and stored at 4 °C for further use.

DSP crosslinking

DSP (1.9 µmol, dissolved in anhydrous DMSO) was added to a suspension of SNP-NH_2 (12 mL, 3.2 mg/mL in 10 mM NaPB, pH 7.5). The reaction mixture was stirred under 400 rpm in a water batch at 20 °C for 20 min. The suspension was then washed twice, sonicated and resuspended in 6 mL of enzyme immobilisation buffer to yield SNP-DSP (6.4 mg/mL).

Glutaraldehyde crosslinking

Glutaraldehyde (Glu, 0.12 mmol, 25% in H_2O) was added to a suspension of SNP-NH_2 (12 mL, 3.2 mg/mL). The reaction mixture was stirred under 400 rpm in a water batch at 20 °C for 30 min. The suspension was then washed twice, sonicated and resuspended in 6 mL of enzyme immobilisation buffer to yield SNP-Glu (6.4 mg/mL).

βgal immobilisation

SNP-DSP (5 mL, 6.4 mg/mL) was mixed with βgal (5 mL, final concentration of 182.6 μg/mL) in 10 mM MES buffer (5 mM $MgCl_2$, pH 6.2). The resulting mixture was stirred under 400 rpm in a water batch at 20 °C for 30 min to produce SNP-DSP-βgal. The resulting nanoparticles were centrifuged at 1500 rcf for 5 min. A BCA assay was carried out on the supernatant collected after enzyme immobilisation, showing that 52.3 % of the enzymes were immobilised at the surface of SNP-DSP (the standard curve generated using the different concentrations of bovin serum albumin (0-2000 μg/mL) is persented at the end of the document; Figure S6).

For immobilisation via glutaraldehyde as a crosslinker, SNP-Glu (5 mL, 6.4 mg/mL) was mixed with βgal (5 mL, final concentration of 180 μg/mL) in 10 mM MES buffer (5 mM $MgCl_2$, pH 6.2). The resulting mixture was stirred under 400 rpm in a water batch at 20 °C for 60 min to produce SNP-Glu-βgal. The resulting nanoparticles were centrifuged at 1500 rcf for 5 min. A BCA assay was performed on the supernatant collected, showing that 52.2% of the enzymes was immobilized at the surface of SNP-Glu.

ASNase immobilisation

SNP-DSP (5 mL, 6.4 mg/mL) was mixed with ASNase (5 mL, final concentration of 14 μg/mL) in 10 mM MES buffer (pH 6.2). The resulting mixture was stirred under 400 rpm in a water batch at 20 °C for 30 min to produce SNP-DSP-ASNase. The resulting nanoparticles were centrifuged at 1500 rcf for 5 min. A BCA assay was carried out, showing that 96% of the enzyme was immobilised at the surface of SNP-DSP.

For immobilisation via glutaraldehyde as a crosslinker, SNP-Glu (5 mL, 6.4 mg/mL) was mixed with ASNase (5 mL, final concentration of 14 μg/mL) in 10 mM MES buffer. The resulting mixture was stirred under 400 rpm in a water batch at 20 °C for 30 min to produce SNP-Glu-βgal. The resulting nanoparticles were centrifuged at 1500 rcf for 5 min. A BCA assay was carried out on the supernatant collected, showing that 87.7% of the enzyme was immobilised at the surface of SNP-Glu.

Enzyme shielding

SNP-DSP-βgal and SNP-Glu-βgal (10 mL, 3.2 mg/mL) were incubated with TEOS (0.134 mmol) in 10 mM ABC buffer (5 mM $MgCl_2$, pH 7.8) at 10 °C, 400 rpm for 1 hour. APTES (0.026 mmol) was subsequently added. The resulting mixture was stirred at 10 °C, 400 rpm for 80 min. Aliquots of 2 mL were collected every 20 min. The resulting nanoparticles were centrifuged at 1500 rcf for 5 min, washed and resuspended in 2 mL MES buffer (5 mM $MgCl_2$, pH 6.2) ([SNP-DSP-βgal-AT] and [SNP-Glu-βgal-AT] = 3.2 mg/mL). The resulting suspension was cured at 20 °C for 16 hours and then stored at 4 °C.
Following the procedure previously described, ASNase was immobilised on particles and shielded with an organosilica layer. The resulting suspension. *i.e.,* SNP-DSP-ASNase-AT and SNP-Glu-ASNase-AT = 3.2 mg/mL, was cured at 20 °C in 10 mM MES buffer (pH 6.2) for 16 hours and then stored at 4 °C.

Scanning electron microscopy characterisation

Particles produced were dispersed in nanopure water and a 2 μL drop of each sample was placed on a silicon substrate. The samples were dried at room temperature under atmospheric conditions and

subsequently sputter-coated with gold for 15 seconds at 20 mA. All samples were imaged using a Zeiss SUPRA® 40VP scanning electron microscope. Secondary electron micrographs were acquired using the InLens mode with an accelerating voltage of 10 kV at a magnification of 150,000 X. The particles' dimensions and layer thickness values were measured using the ImageJ software package. At least 100 particles were measured for each sample.

DTT or GSH treatment

The shieled βgal and shielded ASNase were incubated with DTT (final concentration, 50 mM) for 30 min under 400 rpm in a water batch at 20 °C for 30 min. For the GSH treatment, the shielded ASNase were incubated with L-GSH (final concentration, 1 mM) for 30 min under 400 rpm in a water batch at 20 °C for 30 min. After washing with 10 mM MES buffer trice, the activity assay of the resulting shielded enzyme was performed according to the previously described method.[1] Regarding the activity of soluble ASNase, the enzyme was incubated with L-GSH (with final concentration of 0, 0.1, 0.6, 1 and 5 mM respectively) for 30 min under 400 rpm in a water batch at 20 °C for 30 min. The activity assay was carried out according to the same described method.

SDS-PAGE assay

All samples were firstly washed thoroughly three times with 10 mM ABC buffer (pH 7.8). For the groups reacting with DTT, each of soluble βgal, the immobilised βgal (*i.e.,* SNP-DSP-βgal and SNP-Glu-βgal) and the shielded βgal (*i.e.,* SNP-DSP-βgal-AT after 40, 60 and 80 min of layer growth reaction) samples with equal enzyme amounts was react with 50 mM DTT at 20 °C and 400 rpm for 30 min. 30 µL each sample of the soluble fraction after reaction with DTT and without DTT treatment was mixed with 30 µL of SDS-PAGE sample loading buffer and boiled for 10 min. 10 µL of each resulting mixture was loaded onto 4-20% precast SDS-PAGE gels. The gels were run at 300 V for 30 min and subsequently stained with Coomassie Blue. The gray intensity of bands at ca. 100 kDa in different lanes was obtained using FIJI software and normalized to the group of soluble βgal.

βgal activity assay

The enzymatic activity assay was carried out following the procedure described by M. R. Correro *et al.*[1] O-nitrophenyl-β-D-galactopyranoside (ONPG) was used as substrate. In a typical β-gal activity assay, 1.5 mL of ONPG (21 mM) in KPi buffer (100 mM, 5 mM $MgCl_2$, pH 6.5) were equilibrated at 40°C for 5 min in a thermomixer. 150 µL of soluble βgal, immobilised βgal (*i.e.,* SNP-DSP-βgal and SNP-Glu-βgal) or shielded βgal (*i.e.,* SNP-DSP-βgal-AT, SNP-Glu-βgal-AT with different layer growth durations) were added to the ONPG solution and the mixtures were maintained at 40°C under shaking at 650 rpm. The groups of shielded βgal after reacting with DTT were washed three times with activity buffer before being utilized for the activity assay. Aliquots of 125 µL were collected every 10 minutes and added to 125 µL of 1 M Na_2CO_3 solution to stop the ONPG hydrolysis. The activity is determined by monitoring the amount of the hydrolysed product o-nitrophenol (ONP) at the absorbance of 420 nm in 96 well-plates using Synergy H1 (BioTek). A standard curve is established using ONP to calculate catalytic activity (the standard curve is presented at the end of the document; Figure S7).

ASNase activity assay

ASNase catalyses the hydrolysis of substrate L-asparagine (L-Asn) to generate L-aspartic acid (L-Asp) and ammonia (NH_3)[2]. The enzymatic activity is quantified by measuring of the substrate conversion at the maximum rate, where one unit (U) is defined as the amount of enzyme to convert 1 µmol of L-Asn into 1 µmol of L-Asp and 1 µmol NH_3 per min at 37 °C. The amount of NH_3 produced is measured following

Nessler quantification method established in the literature.[3] The method spectrophotometrically determines the NH_3 produced by reaction with the Nessler reagent dipotassium tetraiodomercurate(II). In a typical assay, 148 µL of phosphate buffer (50 mM, pH 7.4) and 50 µL of L-Asparagine (10 mM) were equilibrate at 37°C for 5 min in a thermomixer. 2 µL of soluble ASNase, immobilised ASNase (SNP-DSP-ASNase, SNP-Glu-ASNase) or shielded ASNase (SNP-DSP-βgal-AT and SNP-Glu-βgal-AT with different layer growth durations) was then added in the substrate solution and incubated at 37°C for 30 min. The sample was added with 50 uL TCA (Trichloroacetic acid, 15%) to stop the hydrolysis of L-Asn and centrifuged at 20,000 g for 3 min. 200 µL of supernatant mixed with 200 µL water and 100 µL Nessler's reagent. The amount of NH_3 produced was determined by measuring the absorbance at 436 nm (OD 436 nm) in a 96 well-plate using Synergy H1. The catalytic activities were calculated using the calibration curve which was prepared through the reaction of Nessler reagent with multiple dilutions of ammonium sulfate ($(NH_4)_2SO_4$) (the standard curve is presented at the end of the document; Figure S8).

Cytotoxicity assay

HepG2 (Hepatoblastoma cell line) cells were cultured in Dulbecco's Modified Eagle Media (DMEM) with high glucose, 10% heat-inactivated fetal bovine serum (FBS) and 1% penicillin-streptomycin under a humidified 5% CO_2 (v/v) atmosphere in an incubator (RS Biotech) at 37 °C. For analysis of cytotoxicity, HepG2 cells were seeded in 96-well plates at a density of 1×10^4 cells per well, which reached 70% confluency after culturing for 72 hours. The cells were then treated with SNP-DSP-ASNase-AT, SNP-Glu-ASNase-AT with identical enzymatic activity and SNP in culture medium without glutamine for 24 hours.

The cytotoxicity assay was carried out using cell counting kit 8 (CCK8; Sigma Aldrich). The kit quantifies the number of live cells by measuring the absorbance of an orange formazan at 450 nm produced by the reduction of cellular dehydrogenases. 10 µl of CCK-8 solution was added into each well and incubated for 2 hours at 37 °C and 5% CO_2 (v/v). The plates were centrifugated at 1500 rcf. 80 µL of the resulting supernatant was taken out and used for absorbance measurement.

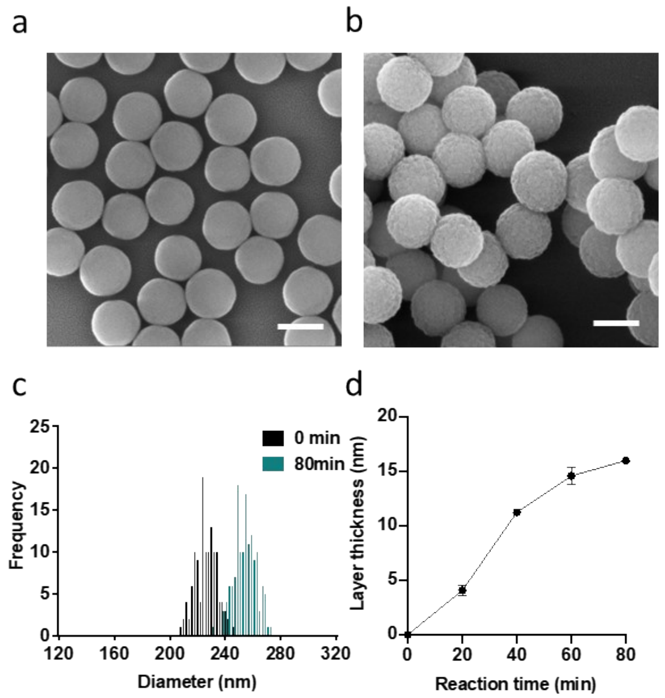

Fig. S1 SEM micrographs of SNPs (**a**) and SNP-Glu-βgal-OS after 80 min of OS layer growth reaction (**b**). Size distribution (**c**) and layer growth kinetics (**d**) measured on SEM micrographs. Every point is the average of at least 100 particles. All scale bars represent 200 nm. Standard error is calculated as $\sigma = ((d_{SNP\text{-}DSP\text{-}\beta gal\text{-}OS} - \bar{d}_{SNPs})/2)/\sqrt{n}$ where n is the number of measured particles.

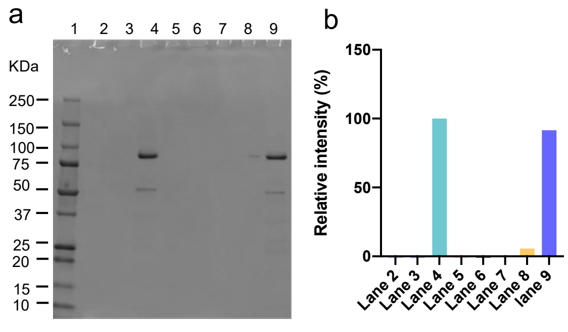

Fig. S2 Effects of DTT treatment on immobilised and shielded βGal cross-linked via DSP and Glu on SNP. (**a**) SDS-PAGE of (*i.e.*SNP-DSP-βGal and SNP-Glu-βGal) with and without DTT treatment. 1- Ladder, 2- SNP-Glu-βGal, 3- SNP-Glu-βGal with DTT treatment, 4- soluble βGal, 5- SNP-DSP-βGal-OS (40 min of layer growth reaction) with DTT treatment, 6- SNP-DSP-βGal-OS (60 min of layer growth reaction) with DTT treatment, 7- SNP-DSP-βGal-OS (80 min of layer growth reaction) with DTT treatment, 8- SNP-DSP-βGal, 9- SNP-DSP-βGal with DTT treatment. (**b**) SDS-PAGE anaylsis of protein bands intensity (*ca*. 100 kDa) in different lanes were obtained using Fiji software.

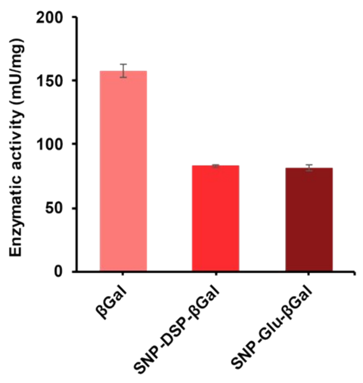

Fig. S3 Enzymatic activity of βgal, SNP-DSP-βgal, and SNPs-Glu-βgal-AT. Error bars represent standard deviation measured on triplicates.

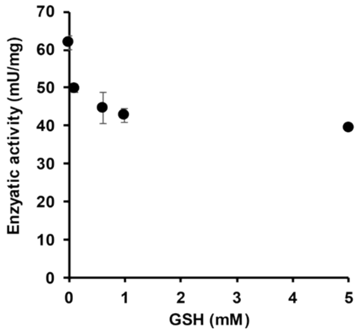

Fig. S4 The activity of soluble ASNase after incubation with glutathione (GSH). Error bars represent standard deviation measured on triplicates.

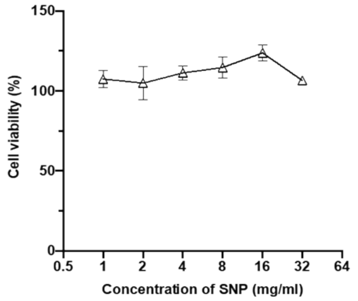

Fig. S5 Cytotoxicity of SNPs. Data are normalized to 100% viability (untreated cells). Error bars represent standard deviation measured on triplicates.

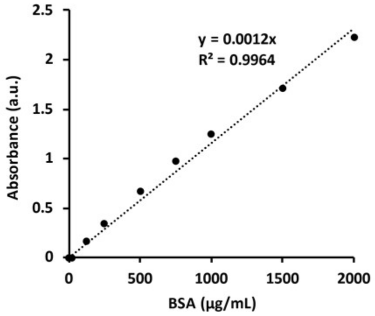

Fig. S6 BCA assay standard curve based on the absorbance of varying concentrations of BSA protein (0-2000 μg/mL).

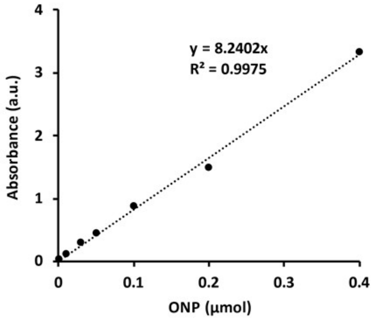

Fig. S7 Standard curve of ONP based on absorbance at increasing unit (0-0.4 μmol).

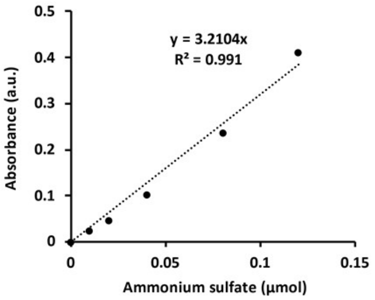

Fig. S8 Standard curve of ammonium sulfate based on absorbance at increasing unit (0-0.12 μmol).

Reference

1 M. R. Correro, N. Moridi, H. Schützinger, S. Sykora, E. M. Ammann, E. H. Peters, Y. Dudal, P. F. X. Corvini and P. Shahgaldian, *Angew. Chemie - Int. Ed.*, 2016, **55**, 6285–6289.

2 N. Verma, K. Kumar, G. Kaur and S. Anand, *Crit. Rev. Biotechnol.*, 2007, **27**, 45–62.

3 J. B. Howard and F. H. Carpenter, *J. Biol. Chem.*, 1972, **247**, 1020–1030.
